# Worries about Being Judged versus Being Harmed: Disentangling the Association of Social Anxiety and Paranoia with Schizotypy

**DOI:** 10.1371/journal.pone.0096269

**Published:** 2014-06-10

**Authors:** Leslie E. Horton, Neus Barrantes-Vidal, Paul J. Silvia, Thomas R. Kwapil

**Affiliations:** 1 University of North Carolina at Greensboro, Greensboro, North Carolina, United States of America; 2 University of Pittsburgh School of Medicine, Pittsburgh, Pennsylvania, United States of America; 3 Universitat Autònoma de Barcelona, Barcelona, Spain; 4 Sant Pere Claver – Fundació Sanitària, Barcelona, Spain; 5 Instituto de Salud Carlos III, CIBERSAM, Barcelona, Spain; Bellvitge Biomedical Research Institute-IDIBELL, Spain

## Abstract

Paranoia is a dimension of clinical and subclinical experiences in which others are believed to have harmful intentions. Mild paranoid concerns are relatively common in the general population, and more clinically severe paranoia shares features with social anxiety and is a key characteristic of schizotypy. Given that subclinical manifestations of schizotypy and paranoia may predict the occurrence of more severe symptoms, disentangling the associations of these related constructs may advance our understanding of their etiology; however no known studies to date have comprehensively evaluated how paranoia relates to social anxiety and schizotypy. The current research sought to examine the association of paranoia, assessed across a broad continuum of severity, with 1) the positive and negative schizotypy dimensions and 2) social anxiety. Specifically, the study tested a series of six competing, *a priori* models using confirmatory factor analysis in a sample of 862 young adults. As hypothesized, the data supported a four-factor model including positive schizotypy, negative schizotypy, social anxiety, and paranoia factors, suggesting that these are distinct constructs with differing patterns of interrelationships. Paranoia had a strong association with positive schizotypy, a moderate association with social anxiety, and a minimal association with negative schizotypy. The results are consistent with paranoia being part of a multidimensional model of schizotypy and schizophrenia. Prior studies treating schizotypy and schizophrenia as homogenous constructs often produce equivocal or non-replicable results because these dimensions are associated with distinct etiologies, presentations, and treatment responses; thus, the present conceptualization of paranoia within a multidimensional schizotypy framework should advance our understanding of these constructs.

## Introduction

The present study examined the relation of paranoia with schizotypy and social anxiety in a non-clinically ascertained sample of young adults. Paranoid disorders are part of the schizophrenia spectrum, and subclinical manifestations of paranoia and suspiciousness frequently occur as part of schizotypy, a continuum of psychotic-like symptoms and impairment that conveys vulnerability for schizophrenia. Paranoia also shares phenomenology with social anxiety. The present study sought to examine the association of paranoia, assessed across a broad continuum of severity, with 1) the positive and negative schizotypy dimensions as well as 2) social anxiety by testing a series of competing models using confirmatory factor analysis (CFA).

### Conceptualizing Paranoia

Paranoia, a common feature of schizophrenia-spectrum disorders, can have profound consequences for social relationships and quality of life. People with paranoia may become socially isolated, and thus less likely to reap the well-known benefits of social support or–when needed–timely referrals by friends and family for clinical intervention. Experiences of paranoia, which range from mild suspiciousness about the intentions of others to firmly entrenched delusions of conspiracy, occur most frequently in schizophrenia-spectrum disorders, but also occur (albeit less frequently) in neurological, mood, and anxiety disorders [1]. There is more to paranoia, however, than its clinical manifestations. Strauss [2] argued that paranoia and other psychotic experiences are best understood as continua, challenging the traditional view that psychotic experiences are categorically distinct from nonpsychotic experiences. Recent studies support this notion, reporting that mild forms of paranoia occur in at least 10% of the general population (e.g., “people are deliberately acting to harm me or my interests”) [3], [4]. For example, Freeman, Garety, Bebbington, Slater et al. [5] found that mild paranoid thoughts occurred in 1/3 of college students. Furthermore, they discovered that extreme paranoid thoughts built hierarchically upon common suspicions, suggesting a continuum of paranoia. Thus, paranoia is not solely a clinical entity, but a continuum of thinking, affect, and behaviors in which others are suspected to have negative and harmful intentions. A better understanding of milder manifestations of paranoia could prove relevant for clarifying the etiology of clinical expressions of paranoia, such as paranoid delusions commonly present in schizophrenia.

### Paranoia and Multi-dimensional Schizotypy

The vulnerability for schizophrenia is expressed across a continuum of clinical and subclinical impairment and is referred to as schizotypy [6], [7], [8], [9]. Schizotypy—and by extension schizophrenia—is thought to be heterogeneous and multidimensional. Studies have identified three dimensions of schizotypy that are parallel to those of schizophrenia: positive symptoms (characterized by disturbances in perceptual experiences and thought content), negative symptoms (characterized by anhedonia, affective flattening, and avolition), and disorganization (characterized by bizarre behavior, thought, and affect) [10], [11]. These dimensions are differentially associated with impairment and risk for psychosis [12], [13], [14], [15]. Multidimensional conceptualizations and measurements of schizotypy and schizophrenia are essential for advancing our understanding of these constructs. Despite this evidence, researchers often treat schizotypy and schizophrenia as homogenous constructs. Studies that treat them as homogenous often produce mixed, equivocal, or non-replicable results because these dimensions are associated with distinct etiologies, presentations, and treatment responses. Given that non-clinical schizotypy predicts the development of psychotic disorders [16], [17], knowledge about the full range of paranoid experiences can assist in understanding etiology and in developing interventions for psychotic and spectrum disorders.

Most factor analytic studies supporting three-factor solutions included paranoia as part of the positive schizotypy symptom dimension, including both studies of people with clinical diagnoses [11] and studies of non-clinical samples [10]. However, recent studies using factor analyses in non-clinical populations have found support for a four-factor model of schizotypy [18], [19], typically consisting of positive, negative, disorganized, and paranoia factors [20], [21]. Most studies have not found a relationship between paranoia and negative schizotypy symptoms. However, Kwapil, Barrantes-Vidal, and Silvia [22] and Kwapil et al. [13] reported that both positive and negative schizotypy dimensions were related to interview ratings of paranoid personality disorder. Conceptually, the ideational component of paranoia (e.g, distorted thinking) fits better with positive schizotypy, whereas the behavioral component (e.g., social withdrawal) fits better with negative schizotypy. However, few studies to date have assessed a broad range of severity and type of paranoid experiences by including multiple measures of paranoia.

### Paranoia and Social Anxiety

Paranoia shares several features with social anxiety, including self-consciousness, social fear, and discomfort with social interaction. Given these similarities, comparing social anxiety and paranoia can clarify the boundaries of paranoia and its place within clinical disorders. A moderate to strong relation of anxiety—both social and general—with paranoia is reported [23], [24], [25]. Studies using non-clinical samples report that paranoid thoughts often build upon relatively common interpersonal worries and anxiety [5], and studies of patients with schizophrenia and spectrum disorders suggest that anxiety may predict the development of paranoia [26], [27].

Researchers have examined the relation of social anxiety with the schizotypy dimensions. Raine et al. [10] initially categorized social anxiety as part of negative schizotypy, but later re-characterized it as part of a third factor known as “disorganization/social impairment” [28]. Brown et al. [29] suggested that social anxiety constitutes a separate factor apart from positive and negative schizotypy; however, social anxiety was more strongly related to positive, rather than negative, schizotypy. This finding is conceptually consistent with additional work suggesting that positive schizotypy is characterized by greater negative affect, including anxiety, whereas negative schizotypy is characterized by less positive affect [14]. No studies to date have comprehensively examined the associations of schizotypy, paranoia, and social anxiety.

### Goals and Hypotheses of the Present Study

The goals of this study were to examine the relation of paranoia with 1) positive and negative schizotypy and 2) social anxiety. The study expanded upon previous research by: (a) employing CFA to compare hypothesis-driven, competing models, (b) examining paranoia and a conceptually similar construct—social anxiety—within the multidimensional framework of schizotypy to address questions not yet resolved in the prior literature (e.g., the relation of paranoia with negative schizotypy), and (c) using multiple measures of schizotypy, social anxiety, and paranoia, thus providing better estimates of these constructs. Six CFA models of increasing complexity were tested. Consistent with Stefanis et al. [20], it was hypothesized that the data would be best described by a four-factor model including positive schizotypy, negative schizotypy, social anxiety, and paranoia, and that the positive schizotypy and paranoia factors would be strongly associated. It was also hypothesized that both positive schizotypy and paranoia would be moderately correlated with social anxiety; however, it was expected that social anxiety and paranoia would not form a coherent “social dysfunction” factor. Negative schizotypy was hypothesized to have minimal association with the other factors.

## Methods

All participants provided written consent. For minors enrolled in this study, written consent was obtained from their guardians/parents on their behalf. The Institutional Review Board at University of North Carolina at Greensboro approved this consent process and all other study procedures.

### Participants

Participants were 862 college students (655 women, 207 men) enrolled in general psychology courses at UNCG. The mean age of the sample was 19.5 years (*SD* = 3.1).

### Materials and Procedures

Participants completed measures as part of departmental mass-screening sessions for course credit. The Revised Social Anhedonia Scale [30] consists of 40 true-false items that tap asociality and indifference to others, and the Physical Anhedonia Scale [31] includes 61 items that measure deficits in sensory and aesthetic pleasure. The anhedonia scales generally tap aspects of negative symptom schizotypy, although the Revised Social Anhedonia Scale is also modestly associated with positive schizotypy [22]. The Perceptual Aberration Scale [32] consists of 35 items that tap perceptual and bodily distortions, and the Magical Ideation Scale [33] contains 30 items that measure implausible beliefs. Groups identified as at-risk by the scales show psychological and physiological deficits similar to those seen in schizophrenia and are at an elevated risk for developing schizophrenia-spectrum disorders [16], [34].

The Schizotypal Personality Questionnaire (SPQ) [35] contains 74 yes-no items that map onto the diagnostic criteria for schizotypal personality disorder. The Suspiciousness (8 items), Ideas of Reference (9 items), and Excessive Social Anxiety (8 items) subscales were used in this study. The Paranoia Checklist [36] is an 18-item scale measuring a range of clinical and non-clinical paranoia. The total score is based upon ratings of frequency, distress, and conviction. The Persecutory Ideas Subscale from Scale 6 of the Minnesota Multiphasic Personality Inventory-Second Edition [37] contains 17 true-false items measuring beliefs that others have harmful intentions. The Social Phobia Scale (SPS) [38] is a 20-item scale that assesses socially phobic concerns of being scrutinized or judged during routine activities.

## Results

Descriptive statistics for the measures are presented in Table 1 and bivariate correlations are presented in Table 2. Alpha level was set at .001 due to the large sample size and the large number of correlations, in order to minimize Type I error, and to reduce the likelihood of reporting statistically significant but inconsequential findings. Consistent with previous findings, the anhedonia scales were significantly correlated, as were the Perceptual Aberration and Magical Ideation Scales. The Revised Social Anhedonia Scale was significantly correlated with the Perceptual Aberration and Magical Ideation Scales, consistent with findings that the scale taps aspects of both positive and negative schizotypy. The measures of social anxiety were positively correlated, as were the measures of paranoia. The paranoia scales were correlated with measures of positive schizotypy, negative schizotypy, and social anxiety. The SPQ-Ideas of Reference subscale was most strongly associated with measures of paranoia, consistent with the self-referential nature of paranoid beliefs.

**Table 1:** Descriptive Statistics for Paranoia, Schizotypy, and Social Anxiety Scales (n = 862).

Paranoia Scales	Mean	SD	Range	Cronbach's α
MMPI- Persecutory Subscale (17 items)	2.64	2.29	0 – 16	.70
Paranoia Checklist (18 items)	32.69	28.49	0 – 196	.88
SPQ- Ideas of Reference (9 items)	3.46	2.47	0 – 9	.75
SPQ- Suspiciousness (8 items)	2.25	1.95	0 – 8	.68
Schizotypy Scales				
Revised Social Anhedonia (40 items)	9.21	5.67	0 – 33	.83
Physical Anhedonia (61 items)	14.28	7.09	0 – 47	.83
Perceptual Aberration (35 items	4.98	4.75	0 – 34	.85
Magical Ideation (30 items)	8.11	5.23	0 – 29	.83
Social Anxiety Scales				
Social Phobia Scale (8 items)	60.30	22.38	15 – 140	.92
SPQ- Excessive Social Anxiety (20 items)	3.62	2.44	0 – 8	.80

Note: SPQ refers to the Schizotypal Personality Questionnaire, MMPI-Persecutory refers to the Minnesota Multiphasic Personality Inventory Version 2 Persecutory Ideas Subscale.

**Table 2:** Correlations of Measures of Paranoia, Schizotypy, and Social Anxiety Scales (n = 862).

	Social Phobia Scale	SPQ-Excessive Soc Anx	Revised Social Anhedonia	Physical Anhedonia	Perceptual Aberration	Magical Ideation	MMPI-Persecutory	Paranoia Checklist	SPQ-Ideas of Reference
SPQ-Excessive Social Anxiety	***0.59*** *								
Revised Social Anhedonia	0.24*	**0.27** *							
Physical Anhedonia	0.08	0.11*	***0.47*** *						
Perceptual Aberration	**0.33** *	**0.25** *	**0.32** *	−0.03					
Magical Ideation	**0.31** *	0.20*	0.22*	−0.14*	***0.68*** *				
MMPI-Persecutory	**0.25** *	0.22*	**0.29** *	0.12*	**0.41** *	***0.45*** *			
Paranoia Checklist	**0.34** *	**0.32** *	**0.29** *	0.08	**0.42** *	**0.41** *	***0.54*** *		
SPQ-Ideas of Reference	**0.30** *	**0.30** *	0.17*	0.00	**0.39** *	***0.55*** *	***0.52*** *	***0.49*** *	
SPQ-Suspiciousness	**0.32** *	**0.37** *	**0.39** *	0.20*	**0.35** *	**0.40** *	***0.65*** *	***0.57*** *	***0.59*** *

*p<.001; Medium effect sizes indicated in bold text, large effect sizes indicated in bold and italicized text.

Note: SPQ refers to the Schizotypal Personality Questionnaire, MMPI-Persecutory refers to the Minnesota Multiphasic Personality Inventory Version 2-Persecutory Ideas Subscale.

To examine the relation of paranoia with social anxiety and schizotypy, six CFAs based upon *a priori* hypotheses were conducted (see Table 3). Both the sample size and number of participants per variable were adequate for conducting CFAs according to recommendations by Bentler and Chou [39]. Following the recommendations of Coffman and McCallum [40], and consistent with Kwapil et al. [22], the items for each of the schizotypy scales were divided into three parcels and the SPS was divided into two parcels. In all models that specified separate positive and negative schizotypy factors, the Revised Social Anhedonia Scale was allowed to cross-load onto both factors, consistent with previous findings [22], [41].

**Table 3:** Summary of Models Tested in Confirmatory Factor Analysis.

Model	Factors	Factor Labels	Scales
Model 1	1	General Distress	Perceptual Aberration, Magical Ideation
			Physical Anhedonia, Revised Social Anhedonia
			Social Phobia Scale, SPQ-Excessive Social Anxiety
			MMPI-Persecutory, Paranoia Checklist, SPQ-Ideas of Reference, SPQ-Suspiciousness
Model 2	2	Schizotypy	Perceptual Aberration, Magical Ideation
			Physical Anhedonia, Revised Social Anhedonia
		Social Dysfunction	Social Phobia Scale, SPQ-Excessive Social Anxiety
			MMPI-Persecutory, Paranoia Checklist, SPQ-Ideas of Reference, SPQ-Suspiciousness
Model 3	2	Positive Schizotypy	Perceptual Aberration, Magical Ideation
			Social Phobia Scale, SPQ-Excessive Social Anxiety
			MMPI-Persecutory, Paranoia Checklist, SPQ-Ideas of Reference, SPQ-Suspiciousness
		Negative Schizotypy	Physical Anhedonia, Revised Social Anhedonia
Model 4	3	Positive Schizotypy	Perceptual Aberration, Magical Ideation
		Negative Schizotypy	Physical Anhedonia, Revised Social Anhedonia
		Social Dysfunction	Social Phobia Scale, SPQ-Excessive Social Anxiety
			MMPI-Persecutory, Paranoia Checklist, SPQ-Ideas of Reference, SPQ-Suspiciousness
Model 5	3	Positive Schizotypy	Perceptual Aberration, Magical Ideation
			MMPI-Persecutory, Paranoia Checklist, SPQ-Ideas of Reference, SPQ-Suspiciousness
		Negative Schizotypy	Physical Anhedonia, Revised Social Anhedonia
		Social Anxiety	Social Phobia Scale, SPQ-Excessive Social Anxiety
Model 6	4	Positive Schizotypy	Perceptual Aberration, Magical Ideation
		Negative Schizotypy	Physical Anhedonia, Revised Social Anhedonia
		Social Anxiety	Social Phobia Scale, SPQ-Excessive Social Anxiety
		Paranoia	MMPI-Persecutory, Paranoia Checklist, SPQ-Ideas of Reference, SPQ-Suspiciousness

Note: SPQ refers to the Schizotypal Personality Questionnaire, MMPI-Persecutory refers to the Minnesota Multiphasic Personality Inventory Version 2-Persecutory Ideas Subscale.

Table 4 reports fit statistics for each of the models. Excellent model fit is indicated by CFI and TLI greater than .95 and RMSEA less than .05 [42]. All chi-square values were significant—as expected given the large sample—so these values were not included in the table. Models were not nested, so change in chi-square could not be compared across successive models to assess improvement in fit. As an alternative method of comparing competing models, the Akaike Information Criterion (AIC) and Browne-Cudeck Criterion (BCC) values were reported. Models with smaller values of AIC and BCC have better fit than competing models; additionally, these fit statistics penalize models with more factors in order to account for the tendency of more complex models to have better fit [43].

**Table 4:** Confirmatory Factor Analyses of Paranoia, Schizotypy and Social Anxiety.

Model	CFI	TLI	AIC	BCC	RMSEA
Model 1	0.74	0.67	2802.68	2804.98	0.13
Model 2	0.76	0.70	2603.79	2606.13	0.12
Model 3	0.77	0.71	2497.61	2499.99	0.12
Model 4	0.81	0.75	2160.23	2162.69	0.11
Model 5	0.92	0.90	1049.93	1052.39	0.07
**Model 6**	**0.96**	**0.94**	**641.44**	**694.01**	**0.05**

Note: Superior fit is indicated by CFI and TLI>.95, RMSEA≤05, smaller values of AIC and BCC.

Consistent with Lewandowski et al. [41] and Brown et al. [29], Model 1 tested whether all scales loaded on a single factor, representing general distress. As indicated in Table 4, this model provided poor fit. Model 2 evaluated the fit of a two-factor model, with one factor, schizotypy, receiving loadings from the schizotypy scales, and a second factor, social dysfunction, receiving loadings from paranoia and social anxiety. This model provided poor fit. Model 3 was an alternative two-factor model with positive schizotypy, including both the paranoia and social anxiety scales, and negative schizotypy factors. This model provided poor fit.

**Figure 1:**
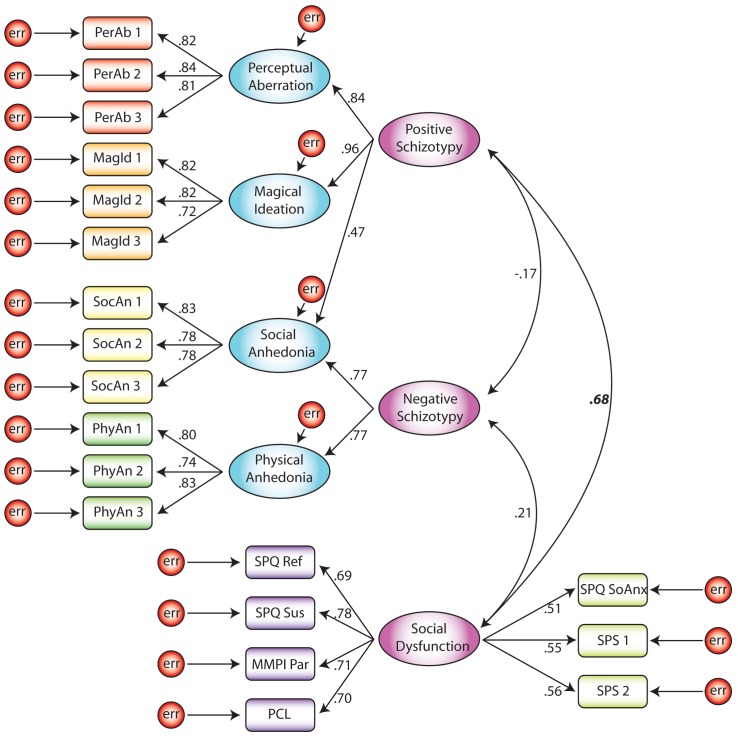
Model 5: Three-factor model with positive schizotypy plus paranoia, negative schizotypy, and social anxiety factors.

Model 4 evaluated a three-factor model consisting of positive schizotypy, negative schizotypy, and a social dysfunction factor that combined social anxiety and paranoia. This model provided poor fit. Model 5 tested an alternative three-factor model with a positive schizotypy factor that included the paranoia scales, a negative schizotypy factor, and a social anxiety factor. This model had adequate to good fit (see Figure 1). Note that one-headed arrows in the figures indicate factor loadings and two headed arrows indicate correlations between factors.

**Figure 2:**
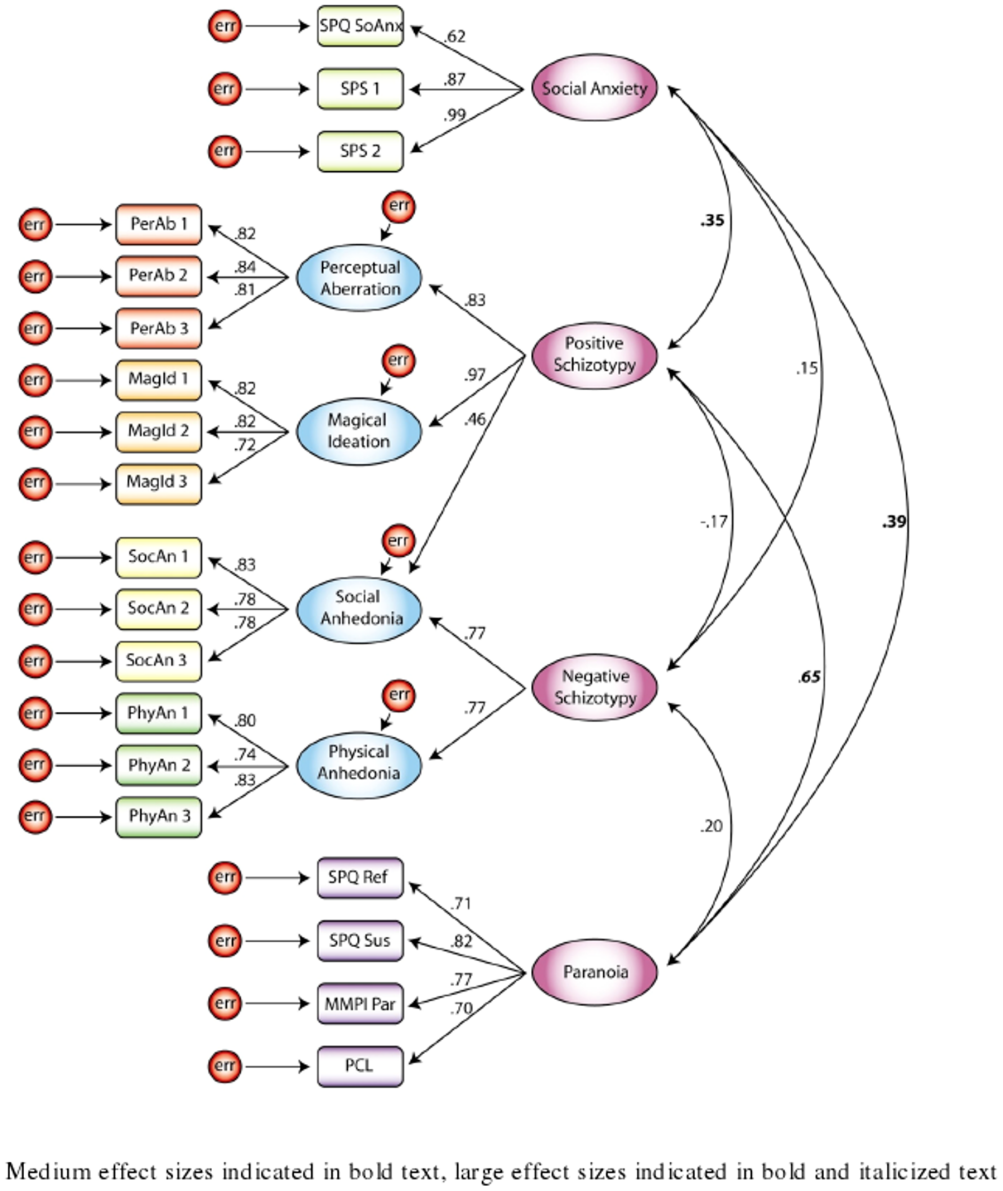
Model 6: Best fitting, four-factor model with positive schizotypy, negative schizotypy, paranoia, and social anxiety factors.

Model 6 examined a four-factor solution consisting of positive schizotypy, negative schizotypy, social anxiety, and paranoia factors (see Figure 2). As hypothesized, this model provided excellent fit and the lowest values of the AIC and BCC. The relationship between the positive schizotypy and paranoia factors represented a large effect size. There was a medium effect for the associations of social anxiety with the positive schizotypy and paranoia factors. The associations of negative schizotypy with the other three factors were small effect sizes.

## Discussion

The present study examined the relation of paranoia with social anxiety, positive schizotypy, and negative schizotypy. The findings are consistent with studies that demonstrated subclinical manifestations of paranoia, and they indicated a wide range of paranoid experiences can be found in non-clinical samples [3]. Thus, these findings support the use of non-clinical samples as a point-of-entry to identify people with suspicious thinking across the range of severity, with particular utility for examining milder forms of suspiciousness that could signal risk for clinical impairment. Clinicians and researchers recognize the importance of improving identification of those at risk for psychotic disorders, as indicated by the addition of “attenuated psychosis syndrome” in Section III (“Area for Further Study”) of the recently published Diagnostic and Statistical Manual for Mental Disorders, 5^th^ edition (DSM-5) [44]. Dimensional assessment of paranoia may be useful in improving identification of those at risk and may allow for a more fine-grained assessment of symptoms across a range of clinical severity. We suggest that creation of a latent paranoia factor from multiple measures provides the best method for assessing the construct; however, if investigators are limited in terms of the number of measures they can include, each of the four measures we used loaded comparably on our paranoia factor.

The present study comprehensively examined the relation of paranoia and schizotypy using CFAs that compared the fit of six models using multiple measures of schizotypy, social anxiety, and paranoia. Consistent with predictions, Model 6—which included positive, negative, social anxiety, and paranoia factors—best fit the data, suggesting that these are distinct constructs with differing patterns of interrelationships.

First, there was a strong correlation between the paranoia and positive schizotypy factors in this model. Note that the self-reference subscale from the SPQ had a high loading on the paranoia factor in Model 6, consistent with other factor analytic studies supporting the inclusion of self-reference with a paranoia factor [20], [21]. Previous research indicates a strong association between cognitive/perceptual aspects of positive schizotypy and paranoia [10], [45]. The present findings support these assertions; however, they also refine our understanding of paranoia as distinct from the cognitive/perceptual aspects of positive schizotypy, consistent with Stefanis et al. [20]. Stefanis et al. noted that several studies reported multiple dimensions of positive symptoms, and that these findings may be minimized in some studies because measures of positive symptoms do not include items specifically tapping paranoia. Furthermore, they noted that the unique perception of the self as threatened, and resulting attempts to compensate for this perception, may account for the divergence of paranoid and self-referential thinking from the cognitive/perceptual distortions characterizing positive schizotypy. This distinction raises the question of whether paranoid delusions have a different origin than other types of delusion in schizophrenia; this issue merits further study and points to the importance of including paranoia measures in future examinations of the structure of schizotypy.

Second, Model 6 found a small relationship between the negative schizotypy and paranoia factors. The few studies prior that have examined the relation of these two constructs found conflicting results [22], [20]. Given the high negative affect and emotional reactivity characterizing paranoia, and the low positive affect and affective flattening characterizing negative schizotypy, a weak relationship between the two seems conceptually consistent. Potential overlap between negative schizotypy and paranoia is likely in the behavioral domain, rather than in the cognitive and affective domains. For example, common measures of both constructs include items about social avoidance. Future studies of paranoia and negative schizotypy should compare ratings on items of behavioral domains to those of cognitive and affective domains.

In addition to examining the relation of paranoia and schizotypy, the present study examined the relation of social anxiety to paranoia. Paranoia and social anxiety were found to be distinct constructs. Consistent with previous findings [29], paranoia and social anxiety were more strongly related to positive than to negative schizotypy and were moderately related to one another. The overlap between features of paranoia and social anxiety, such as social discomfort and heightened self-awareness, account for the moderate relationship between paranoia and social anxiety and are consistent with the literature [23]. Furthermore, the differences between paranoia and social anxiety explain the poor fit of models combining the two constructs in the present study (notably in Model 4). Paranoia is characterized by a lack of trust in the motives of others and hostility; social anxiety is characterized by a lack of trust in one's own ability to meet social demands and self-blame. More studies are needed to understand how these constructs relate. If clinical paranoia is an antecedent of mild suspicious concerns, as suggested by Freeman, Garety, Bebbington, Slater et al. [5], examining the range of paranoid experiences in typical people, and its relation to conceptually similar and common experiences of social anxiety and schizotypy, may help us understand the developmental trajectory of how suspiciousness develops into clinical symptoms such as paranoid delusions. For example, future studies could examine whether the experience of feeling self-conscious and anxious are necessary precursors to paranoia.

An implication of these findings is that future studies of paranoia, social anxiety, and schizotypy should consider the motives behind social isolation, given a lack of clarity about the nature of social behaviors has contributed to a poor consensus on the nature of symptoms in the literature. For example, previous factor analytic studies of the schizophrenia spectrum have identified a third factor labeled variously as “disorganization” and a “disorder of relating”; in some factor analytic studies, paranoia and social anxiety comprise part of a positive schizotypy factor, and, in others, they are considered a part of negative schizotypy.

To illustrate how failing to consider motives for social dysfunction contributes to conceptual confusion, consider a hypothetical item: “I am alone more often than other people.” Agreement could be due to a preference for solitude due to a lack of positive reinforcement from social contact (negative schizotypy), a fear of being judged or criticized by others (social anxiety), an avoidance of contact due to embarrassment about perceptual anomalies (positive schizotypy), or a belief that others will harm them (paranoia). Failing to account for these different interpretations of social behavior can hinder the progress of research on the schizophrenia spectrum.

We suggest that experience sampling methodology or ecological momentary assessment provides a powerful tool for examining the expression of paranoid experiences and disentangling these experiences from social anxiety. Recent studies in clinical and non-clinical samples [46], [26], [47] offer promising examples of how these daily life research tools can tease apart the associations between these related constructs, the temporal architecture of these experiences in the real world, and the contextual factors that impact the likelihood of momentary paranoia.

As noted in the introduction, numerous models suggest schizotypy and schizophrenia include a cognitive and behavioral disorganization dimension. The fact that we did not model this dimension in our CFAs was not meant to indicate we do not believe this dimension is part of schizotypy, but rather reflects that the measures we included in our study simply do not tap this dimension. Future studies should examine the role of disorganization in schizotypy and its relations with the other dimensions. However, Kwapil et al. [22] pointed out that questionnaires have not been entirely successful measuring mild forms of formal thought disturbance and behavioral disorganization. Furthermore, Gross et al. [48] stated such measures may in fact be tapping oddity associated with positive schizotypy, not actual disorganization.

The present findings suggest the accurate screening of paranoia, social anxiety and schizotypy across the spectrum of impairment will assist in improved differential diagnosis and identification of those at risk for psychosis. Prior evidence supports that positive and negative schizotypy dimensions are associated with distinct etiologies, presentations, and treatment responses, and the present conceptualization of paranoia and social anxiety within the schizotypy dimensions should advance our understanding of these differences. Implications for cognitive-behavioral treatment of psychosis, for example, may include the need for more comprehensive evaluation of paranoia and anxiety in order to more effectively treat their behavioral outcomes, such as social withdrawal. Thus, better assessment of these paranoia and the schizotypy dimensions could provide more specific information about which behaviors to target in future treatment and prevention efforts for populations at risk for psychotic disorders.
